# Integrative Genomic and Transcriptomic Analysis in Acute Interstitial Pneumonia

**DOI:** 10.1111/jcmm.70252

**Published:** 2024-12-05

**Authors:** Donghee Kwak, Junho Kang, Yeuni Yu, Hansong Lee, Yeongjoo Kim, Eun Jung Kwon, Dong Min Lim, Seongik Mun, Hyo Min Kim, Hae Seul Lee, Yun Hak Kim, Hye Ju Yeo, Woo Hyun Cho

**Affiliations:** ^1^ Convergence Medical Sciences Pusan National University Yangsan Republic of Korea; ^2^ Department of Research Keimyung University Dongsan Medical Center Daegu Republic of Korea; ^3^ Medical Research Institute Pusan National University Yangsan Republic of Korea; ^4^ Interdisciplinary Program of Genomic Data Science Pusan National University Yangsan Republic of Korea; ^5^ Department of Biomedical Informatics, School of Medicine Pusan National University Yangsan Republic of Korea; ^6^ Department of Anatomy, School of Medicine Pusan National University Yangsan Republic of Korea; ^7^ Department of Internal Medicine, School of Medicine Pusan National University Yangsan Republic of Korea; ^8^ Division of Pulmonary, Allergy, and Critical Care Medicine, Department of Internal Medicine Pusan National University Yangsan Hospital Yangsan Republic of Korea; ^9^ Transplantation Research Center, Research Institute for Convergence of Biomedical Science and Technology Pusan National University Yangsan Hospital Yangsan Republic of Korea

**Keywords:** acute interstitial pneumonia, expression quantitative trait loci, transcriptomics, whole genome sequencing

## Abstract

Acute Interstitial Pneumonia (AIP) represents a severe form of diffuse lung injury within the idiopathic interstitial pneumonia spectrum. Given the limited understanding of its molecular basis, this study aims to elucidate AIP's genomic and transcriptomic profiles to uncover its pathophysiological underpinnings and identify potential therapeutic targets. We conducted a comprehensive analysis of genomic and transcriptomic data from lung tissues of 15 AIP patients. This included assessing differentially expressed genes (DEGs) and identifying mutations in exonic coding variants, as well as analysing expression quantitative trait loci (eQTL) profiles to link non‐coding SNP genotypes with gene expression levels. Transcriptomic analysis revealed a significant upregulation of genes linked to the Type I interferon receptor and keratin filament, and a downregulation of genes related to focal adhesion and endothelial integrity, compared to healthy individuals. These patterns were distinct from those observed in idiopathic pulmonary fibrosis (IPF) and non‐IPF interstitial lung diseases (ILDs). Genomic analysis highlighted mutations in genes associated with keratin and the extracellular matrix. Additionally, eQTL profiling provided insights into the genetic regulation of gene expression in AIP. Our findings reveals AIP's unique molecular landscape, differentiating it from other ILDs and laying the groundwork for future diagnostic and therapeutic research.

## 1 Introduction

Acute interstitial pneumonia (AIP), synonymous with Hammon‐Rich syndrome, is an idiopathic interstitial pneumonia characterised by acute onset, rapidly progressive, and distinct from chronically progressive idiopathic pulmonary fibrosis. Its pathologic hallmark is diffuse alveolar damage, leading to severe and diffuse lung injury and rapidly progressive acute respiratory failure, particularly in patients without prior lung disease. This rapid progression sets it apart from other interstitial lung diseases, underscoring its clinical and pathological [1, 2]. Diagnosis of AIP is challenging, and it is essential to rule out other causes, such as other interstitial lung diseases, infection, and diffuse alveolar haemorrhage.

The management of AIP primarily relies on supportive care, as there is no established treatment. Ensuring adequate oxygenation is a critical challenge, often necessitating mechanical ventilation and or extracorporeal membrane oxygenation (ECMO). Initially, broad‐spectrum antibiotics are administered until infectious causes are excluded. Steroid therapy, although commonly used, has not demonstrated clear benefits in AIP. Its efficacy remains uncertain, as evidenced by varying survival rates in different case studies. For patients who do not respond to conventional treatments, a palliative therapeutic option like lung transplantation is considered.

Currently, little is known about the pathogenesis of AIP. Unknown damage to the alveolar epithelium is thought to activate an inflammatory cascade, ultimately leading to fibroblast activation, fibrosis, and lung injury. Unfortunately, AIP is difficult to diagnose and treat clinically, and the patient's condition is so severe that tissue is challenging to obtain, which has many limitations in research and was mainly limited to research using postmortem tissue. In this context, a more in‐depth understanding of the triggers and progression of AIP is needed to improve patient outcomes and develop new treatment options [3], and extensive genomics and transcriptomics research are required [4]. Recently, expression quantitative trait locus (eQTL) mapping effectively links disease‐associated genetic variants with gene regulatory mechanisms [5]. This approach was particularly significant in lupus, where they proposed a pharmacogenetic strategy to explore drug‐gene interactions, utilising eQTL analysis to understand and potentially mitigate the disease's impact [6]. This study aimed to uncover the molecular mechanisms of AIP, a condition yet to be fully understood, by employing comprehensive genomic and transcriptomic analysis, including eQTL analysis using lung tissue from AIP.

## 2 Methods

### 2.1 Study Participants and Data Collection

RNA sequencing and Whole genome sequencing (WGS) were performed on paraffin blocks obtained from explanted native lungs (AIP, *n* = 15) of lung transplant recipients and from normal control subjects (donor lungs, *n* = 5). The Pusan National University Yangsan Hospital (PNUYH) Biobank distributed lung tissue samples from anonymized lung transplant recipients with AIP (*n* = 15) and lung tissue samples from healthy donor lungs (*n* = 5) obtained after lung volume reduction. The diagnosis of AIP was decided after a multidisciplinary meeting of pathologists, pulmonologists and radiologists by combining imaging findings, clinical course and pathological findings [7]. All studies were approved by the Institutional Review Board of Pusan National University Yangsan Hospital (PNUYH IRB No 55‐2024‐002). All tissues from the biobank were anonymized, and the need for informed consent was waived. All methods were performed in accordance with the relevant guidelines and regulations.

### 2.2 RNA Extraction and Library Preparation

RNA was extracted from cell samples using the TRIzol reagent (Invitrogen) and assessed for integrity. RNA libraries were prepared using the Illumina TruSeq Stranded Total RNA Library Prep Gold Kit and sequenced on an Illumina NovaSeq platform. The RNA concentration and quality were measured with the Quant‐IT RiboGreen and TapeStation RNA screentape, respectively. For library construction, 100 ng of RNA was fragmented, converted to cDNA, and subjected to end repair and adapter ligation. The Agilent SureSelect XT Human All Exon v6 + UTRs Kit was used for exonic region capture, followed by PCR amplification. The final library was quantified using qPCR and qualified on the TapeStation D1000. Sequencing was performed by Macrogen Inc. on the NovaSeq system, generating paired‐end reads.

### 2.3 DNA Extraction and Library Preparation

Genomic DNA was extracted from 20 to 40 mg samples of freshly frozen lung tissue using the PureLink Genomic DNA Mini Kit (K182000, Invitrogen, Waltham, MA, USA). This process adhered to the guidelines provided by the manufacturer. The concentration of total DNA was measured using the QuantiFluor dsDNA System (E2671, Promega, Madison, WI, USA). The integrity of the genomic DNAs (gDNAs) was evaluated using the Genomic DNA ScreenTape Analysis system (5067‐5366, Agilent, Santa Clara, CA, USA) in conjunction with the 4200 TapeStation System (G2991BA, Agilent, Santa Clara, CA, USA). The sequencing libraries were constructed following the protocol provided with the TruSeq Nano DNA High Throughput Library Prep Kit (20015965, Illumina Inc., San Diego, CA, USA). Paired‐end sequencing data (2 × 150 bp) were produced using the NovaSeq system (20012850, Illumina Inc., San Diego, CA, USA), achieving target depths of 30× and 60× for different samples.

### 2.4 Data Preprocessing of the RNA Sequencing

Quality assessment of paired‐end reads from the Illumina sequencer was performed using FastQC (v0.11.9) [8]. Reads exhibiting a Phred score above 35 were classified as high quality. Adapter sequences were eliminated using Cutadapt [9], specifically removing “AGATCGGAAGAGCACACGTCTGAACTCCAGTCA” from the forward strand and “AGATCGGAAGAGCGTCGTGTAGGGAAAGAGTGT” from the reverse strand. We aligned the processed reads to the *Homo sapiens* (hg38) genome sequence using HISAT v2.1.0 [10]. After alignment, we employed featureCounts v1.6.0 [11] to generate a transcript count table for each sample, which was utilised for subsequent analyses.

### 2.5 Analysis of RNA Sequencing

Raw counts were transformed into log counts per million using the calcNormFactors() and cpm() functions from the edgeR package [12], after genes not expressed were filtered out using filterByExpr(). Differential expression analysis was carried out through linear modelling and Bayes moderation, post‐adjusting for heteroscedasticity in count data with voom(). Gene Ontology analysis of differentially expressed genes (DEGs) was performed using Clusterprofiler v4.6.2 [13].

### 2.6 Data Preprocessing of the WGS Data

We performed quality checks on paired‐end reads generated by the Illumina sequencer using FastQC, considering reads with a Phred score greater than 35 as high quality. Adapter sequences were also removed using Cutadapt. For read alignment, BWA‐MEM (v0.7.15) [14] was utilised to map the reads to the hg38 human reference genome, with the mapped reads subsequently converted to BAM format using Samtools [15]. Duplicate reads were eliminated using MarkDuplicatesSpark from GATK (v4.2.4.1). To assess base call accuracy, we employed GATK's BaseRecalibrator, which generated base quality scores, using dbSNP_146, hapmap_3.3, and the 1000 Genomes databases for base quality score recalibration. Systematic errors introduced by the sequencing machine were identified and corrected using ApplyBQSR.

### 2.7 Somatic Variant Calling

Somatic variant calling in this study was conducted following the GATK best practices for short somatic variant discovery. The analysis involved whole‐genome sequences. For calling SNV, Mutect2 was used, leveraging local assembly of haplotypes within the active region. Utilising a normal panel generated from participants in the 1000 genomes project, the analysis also incorporated the gnomAD database as a “germline‐resource” to identify somatic variants. FilterMutectCalls was utilised to eliminate correlated errors. Following variant calling, each variant was annotated using Funcotator.

### 2.8 Analysis of Somatic Variation

The mutation annotation file, generated after variant calling and annotation, was imported into R using the maftools [16]. With this package, we identified the variant types in our samples, determined the top mutated genes, and displayed the proportion of samples impacted by these genes. For further analysis, we focused on genes with somatic variants exhibiting a mutation rate of over 20% and conducted GO analysis of these genes using Clusterprofiler. Particular attention was given to genes annotated in GATK funcotator with gnomAD_exome_AF_eas_kor values at or below 0.05.

### 2.9 Analysis of eQTLs

eQTLs were detected by the association analysis of genotypes and gene expression using Matrix eQTL v2.3 [17]. In this study, non‐coding SNPs with a mutation rate of 20% or higher among 15 patients were used to determine genotype using SNPs identified with Mutect2. The log counts per million table was used as expression data. ggplot2 v3.4.4 was used to show manhattan plot of SNPs corresponding to eQTL for each chromosome and change in the expression profile associated with genotypes. We used ggplot2 to perform regression analyses on changes in expression profiles related to genotype using eQTLs (false discovery rate (FDR) ≤ 0.05).

## 3 Results

### 3.1 Overall Study Design

**FIGURE 1:**
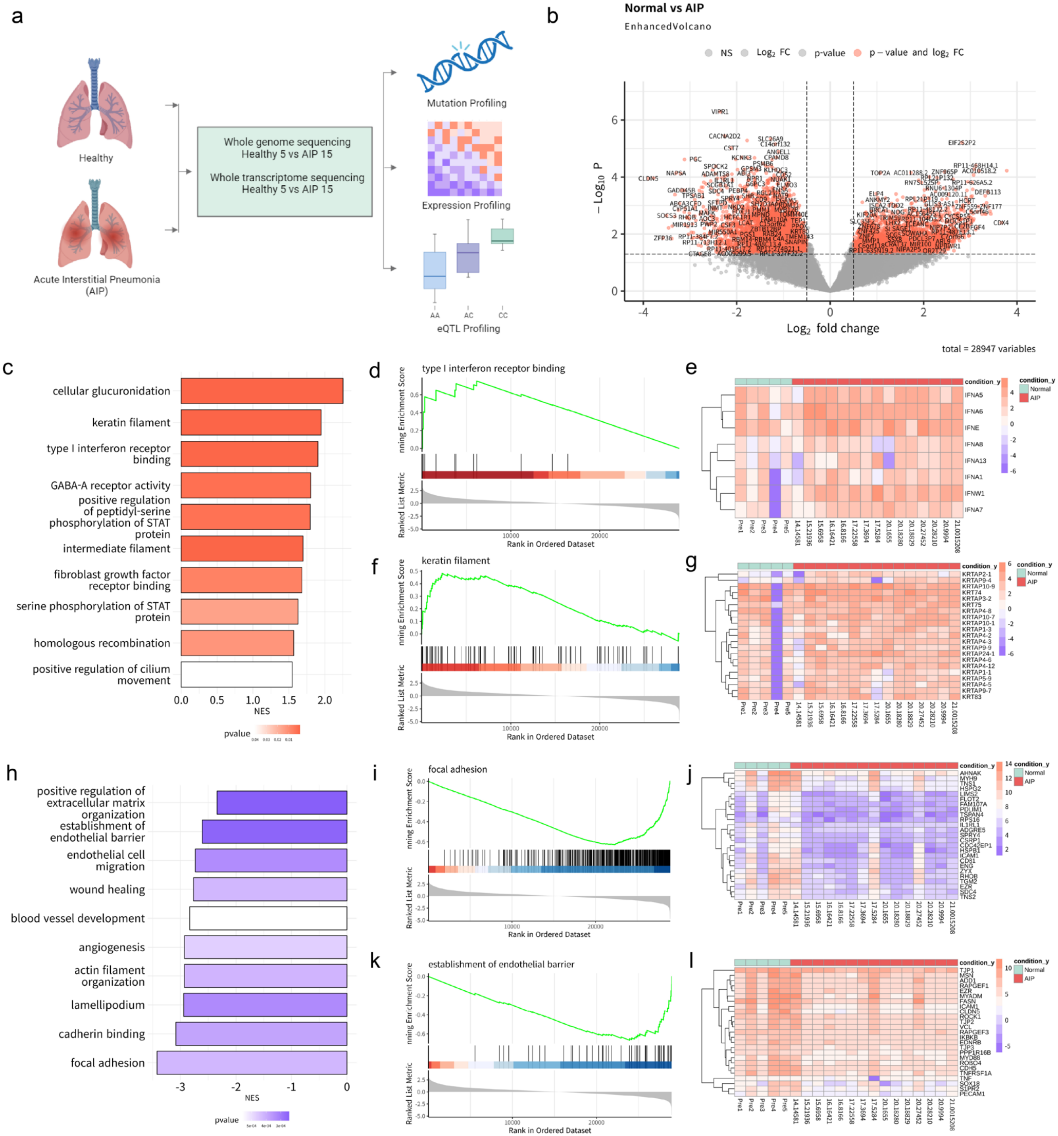
Summary of this study and transcriptomic profiling of AIP. (a) Analysis scheme on this study. (b) Volcano plot depicting the dysregulated mRNAs of AIP RNA sequencing. (c) Curated enriched GO terms of the differentially upregulated genes were listed in the bar plot. (d) GSEAplot confirmed GO term: Type I interferon receptor binding was enriched. (e) Heatmap of DEGs expression which in GO term: Type I interferon receptor binding. (f) GSEAplot confirmed GO term: Keratin filament was enriched. (g) Heatmap of DEGs expression which in GO term: Keratin filament. (h) Curated enriched GO terms of the differentially downregulated genes were listed in the bar plot. (i) GSEAplot confirmed GO term: Focal adhesion. (j) Heatmap of DEGs expression which in GO term: Focal adhesion. (k) GSEAplot confirmed GO term: Establishment of endothelial barrier was enriched. (l) Heatmap of DEGs expression which in GO term: Establishment of endothelial barrier.

The participants in this study were 5 healthy controls and 15 AIP patients (Tables S1 and S2). The median age of AIP patients was 55 years, with 46.7% male. Most (80%) were never smokers, and 20% were former smokers (Table S1). The study's aim was to identify the key molecular features associated with AIP. For this purpose, we conducted multi‐omics sequencing on lung tissue samples. This included whole‐genome sequencing (WGS) and RNA sequencing (RNA‐seq) for both the AIP patient samples (*n* = 15) and a control group of healthy individuals (*n* = 5). In this study, we have conducted an in‐depth exploration of transcriptomics and genomic profiling to gain insights into AIP, as well as investigating potential expression quantitative trait loci (eQTLs) (Figure 1a).

### 3.2 Transcriptomic Analysis of AIP

We investigated the altered gene expression profiles in AIP using RNA‐seq data from 5 healthy controls and 15 AIP patient samples. We identified 3046 upregulated (log_2_ fold change > 0.5, *p* < 0.05) and 3703 downregulated DEGs (log fold change < −0.5, *p* < 0.05) (Figure 1b and Table S3). These genes were significantly enriched in several pathways, including keratin filament, intermediate filament, type I interferon receptor binding, STAT protein phosphorylation, GABA‐A receptor activity, and fibroblast growth factor receptor binding (Figure 1c). A significant enrichment of upregulated genes in the AIP group suggests that type I interferon receptor binding and keratin filament pathways were indeed activated in these patients (Figure 1d,f). Hierarchical clustering of core genes, the subset of genes contributing most to the enrichment result, revealed the most overexpressed AIP‐associated DEGs, notably interferon‐alpha and keratin‐associated genes (Figure 1e,g). On the other hand, downregulated genes were significantly enriched in pathways such as focal adhesion, angiogenesis, establishment of endothelial barrier, and positive regulation of the extracellular matrix (Figure 1h). The significant enrichment of downregulated genes in the AIP group suggests dysregulation in the focal adhesion and establishment of the endothelial barrier (Figure 1i,k). Hierarchical clustering of core genes revealed the most under‐expressed AIP‐associated DEGs, notably those involved in the positive regulation of the extracellular matrix and establishment of the endothelial barrier (Figure 1j,l).

### 3.3 Distinct Expression of AIP Differentiating From IPF and Non‐IPF ILDs

**FIGURE 2:**
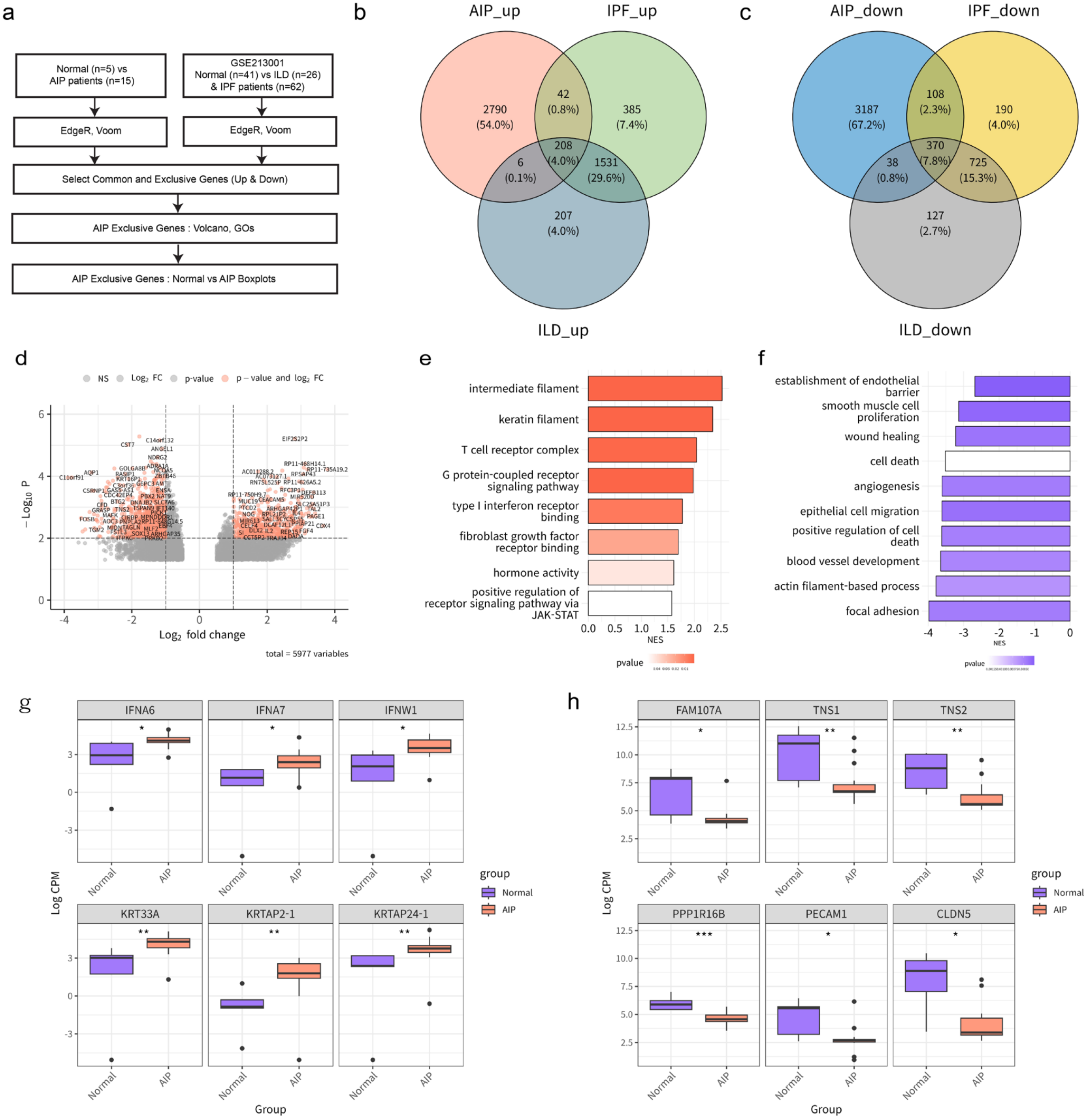
Analysis of AIP‐specific expression markers compared to IPF and non‐IPF ILD (a) Study workflow for select AIP specific DEGs containing the steps of data analysis. (b) Venn diagram of genes upregulated in AIP, IPF and ILD. (c) Venn diagram of genes downregulated in AIP, IPF and ILD. (d) Volcano plot depicting the AIP specific DEGs. (e) The enriched GO terms derived from the differentially upregulated genes specific to AIP were catalogued and displayed in the bar plot. (f) The enriched GO terms derived from the differentially downregulated genes specific to AIP were catalogued and displayed in the bar plot. (g) Group Wilcoxon test comparison of *IFNA6*, *IFNA7*, *IFNW1*, *KRT33A*, *KRTAP2‐1*, *KRTAP24‐1* (h) Group Wilcoxon test comparison of *FAM107A*, *TNS1*, *TNS2*, *PPP1R16B*, *PECAM1*, *CLDN5* (*p* value < 0.001: ***, *p* value < 0.01: **, *p* value < 0.05: *, non‐significant: ns).

AIP is distinguished from idiopathic pulmonary fibrosis (IPF) within the spectrum of interstitial lung diseases (ILD) and is categorised as Idiopathic Interstitial Pneumonia (IIP) [2]. However, specific molecular markers that distinctly identify AIP from other ILDs have not yet been established. Consequently, this study aims to identify unique markers exclusively expressed in AIP by analysing public GEO data (GSE213001), which includes samples from both IPF and non‐IPF ILDs (Figure 2a). Analysis of selected transcriptomic datasets identified sets of DEGs in AIP compared to IPF and non‐IPF ILDs. Further analysis revealed 2790 and 3187 unique DEGs exclusive to AIP, hereafter referred to as ‘AIP‐specific genes’ (Figure 2b,c). GO analysis was conducted on these 2790 upregulated and 3187 downregulated AIP‐specific genes (Figure 2d). The results indicated that the significant functions identified were largely consistent with those observed in Figure 1 (Figure 2e,f). We employed a Wilcoxon test to verify the statistical significance of expression differences in AIP‐specific core genes between normal and AIP groups, as identified by Gene Ontology (GO) (Figures S1 and S2). Among the upregulated AIP‐specific core genes, *IFNA6*, *IFNA7*, *IFNW1* in the type I interferon receptor binding, *KRT33A*, *KRTAP2‐1*, *KRTAP24‐1* in intermediate filament, all showed significant differences (Figure 2e). Conversely, the downregulated AIP‐specific core genes—*FAM107A*, *TNS1*, *and TNS2* in focal adhesion, along with *PPP1R16B*, *PECAM1*, and *CLDN5* in the establishment of the endothelial barrier—also exhibited significant differences between the two groups (Figure 2f).

### 3.4 The Genomic Alteration of AIP

**FIGURE 3:**
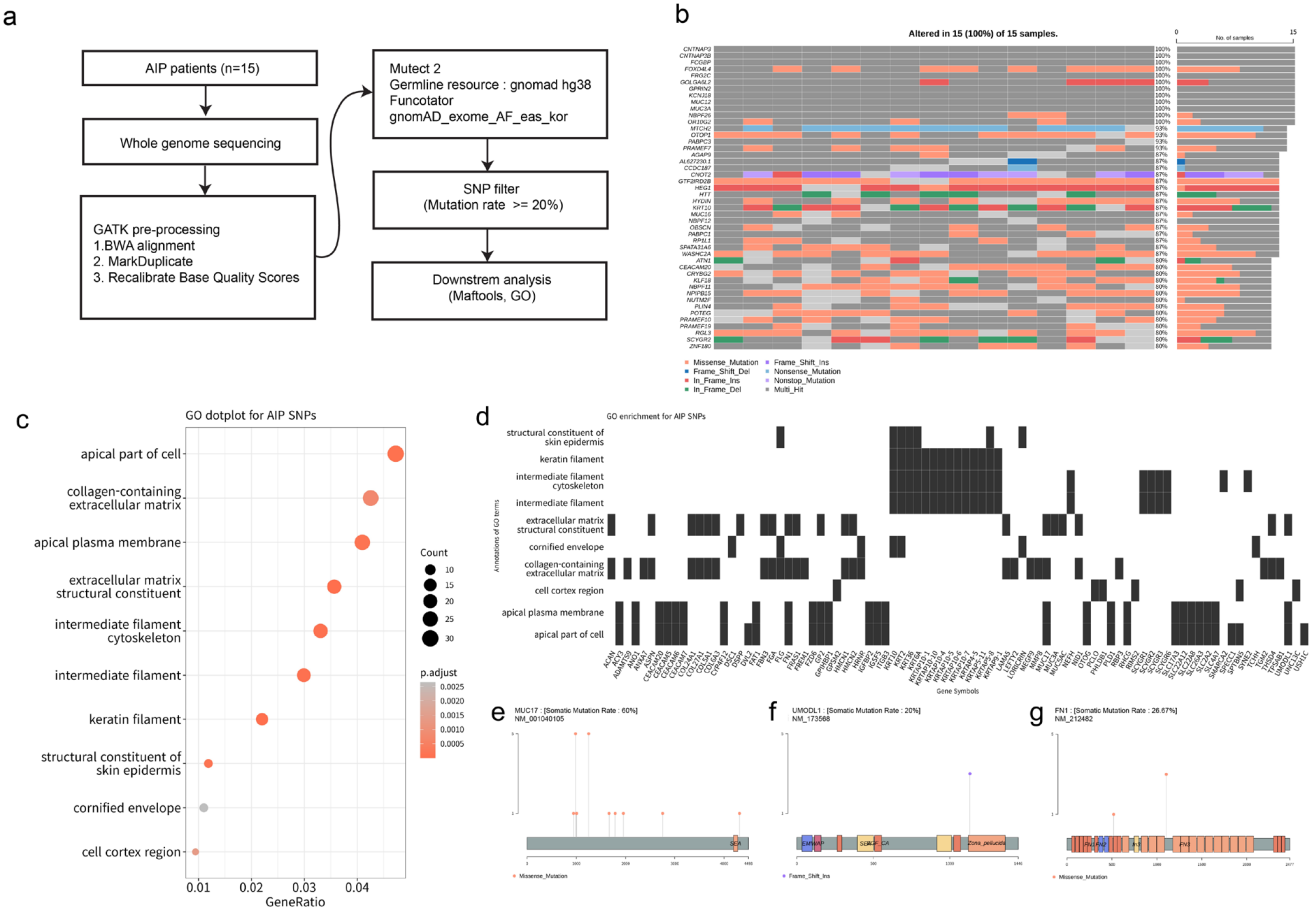
Somatic variants profiling of AIP. (a) Schematic workflow of WGS in AIP. (b) Mutation profiling of AIP patients. (c) GO Analysis highlighting SNPs present in three or more patient samples. (d) Correspondence of Gene Ontology (GO) terms with individual genes. (e–g). Distribution of *MUC17*, *UMODL1* and *FN1* mutations.

We analysed WGS data from 15 AIP patients. After preprocessing and applying Mutect2 for variant filtering, we identified a total of 10,781 coding variants. To focus on more commonly occurring genetic variations, we selected variants present in at least three out of the 15 samples examined. This criterion led to the selection of 1082 variants for further analysis (Figure 3a). The mutation profiling represents the top 46 mutated genes across the 15 samples (Figure 3b). The most frequently mutated genes in our AIP patients were *CNTNAP3*, *CNTNAP3B*, *FCGBP*, *FOXD4L4*, *FRG2C*, *GOLGA6L2*, *GPRIN2*, *KCNJ18*, *MUC12*, *MUC3A*, *NBPF26*, *and OR10G2*. Subsequently, we conducted a comprehensive gene ontology analysis on 721 genes harbouring the 1082 coding variants (Figure 3c). Notably, functions related to intermediate filament, keratin and ECM, previously identified in the transcriptome analysis (Figure 1c,h), were also detected. Additionally, variants were observed in genes associated with a diverse array of biological structures, including the apical part of the cell, apical plasma membrane, collagen‐rich extracellular matrix, and structural constituents of the extracellular matrix. Genes enriched in various GO terms were delineated (Figure 3d). Functions related to keratin filament and intermediate filament showed recurrent correspondence with genes such as *KRT* and *KRTAP*. Additionally, genes aligned with functions pertaining to the extracellular matrix structural constituent and collagen‐containing extracellular matrix. Among the genes linked to three or more GO terms, amino acid changes in *MUC17* (2.87175e‐02), *UMODL1* (4.87166e‐02) and *FN1* (4.30528e‐02) were highlighted (Figure 3e–g). These genes, annotated in GATK funcotator with gnomAD_exome_AF_eas_kor values of 0.05 or below, received specific focus. We observed that *MUC17* displayed various missense mutations, among which T983N and P1249A were each observed in five patients. In *UMODL1*, a frameshift insertion, Q1131fs, occurred in three patients, while in *FN1*, the missense mutation I1103L was identified in three samples. We hypothesise that these nonsynonymous mutations could elucidate the pathogenesis in AIP patients.

### 3.5 The eQTL Profiling of AIP

**FIGURE 4:**
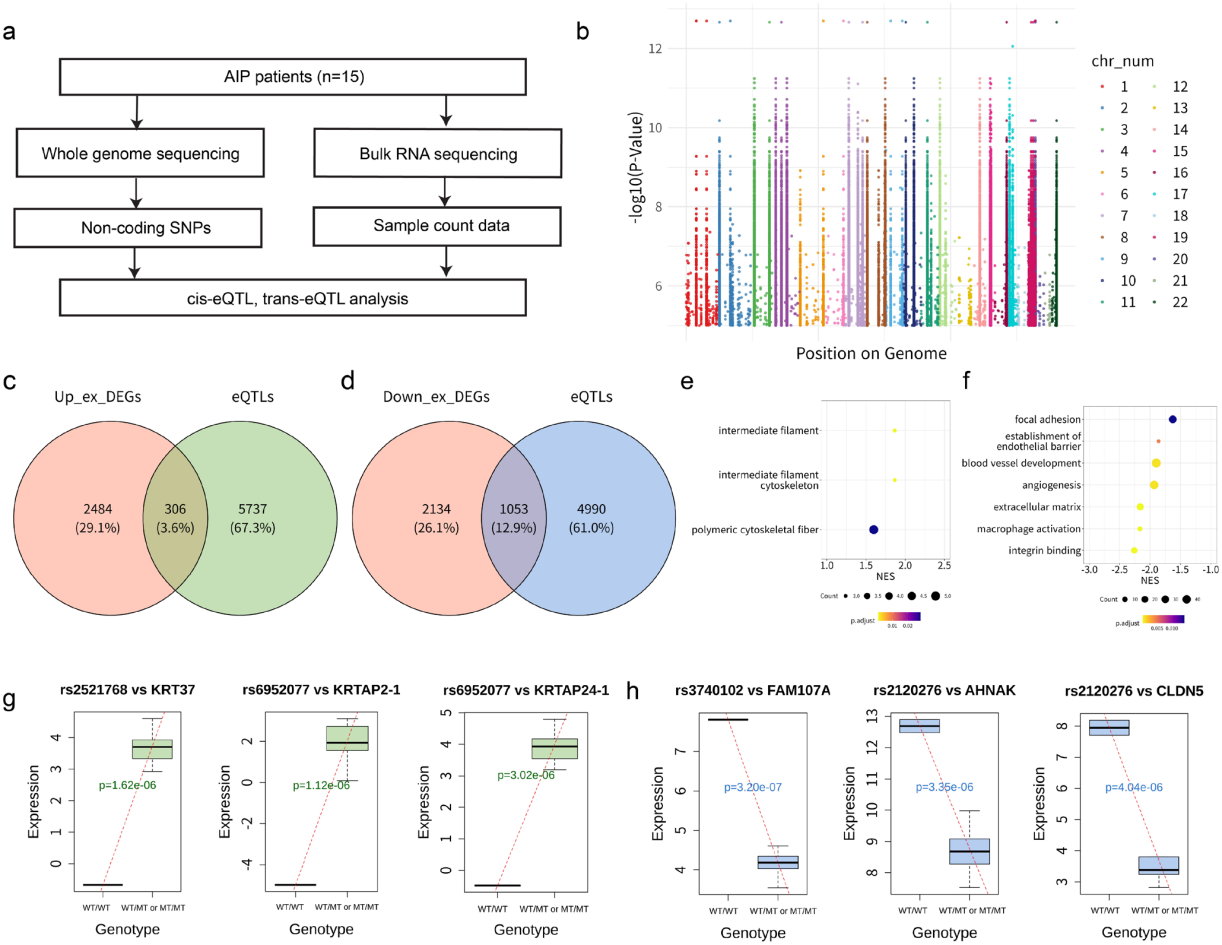
eQTLs profiling of AIP. (a) Schematic workflow of expression quantitative trait loci (eQTL) in AIP. (b) Manhattan plot of genome‐wide distribution of the eQTLs. (c) Shared genes between upregulated AIP‐specific genes and eQTL‐affected genes. (d) Shared genes between downregulated AIP‐specific genes and eQTL‐affected genes. (e) GO analysis of common genes shown in c. (f) GO analysis of common genes shown in d. (g) eQTL analysis depicting genotype‐expression regression of genes associated with intermediate filament function. (h) eQTL analysis depicting genotype‐expression regression of genes associated with down regulated functions in AIP.

We performed eQTL analysis on AIP patients, using methodologies detailed in the ‘Methods’ section (Figure 4a). The association *p* values of multiple SNPs on autosomal chromosomes for all cis and trans analyses are illustrated in Manhattan plots (Figure 4b). We identified common sets between upregulated AIP‐specific DEGs and eQTLs, as well as between downregulated AIP‐specific DEGs and eQTLs, and proceeded with further analysis (Figure 4c,d). The common set for upregulated AIP‐specific DEGs and eQTLs comprised 306 genes, while the set for downregulated AIP‐specific DEGs and eQTLs included 1056 genes. Subsequently, we conducted a GO enrichment analysis for these two gene groups (*p* value < 0.05) (Figure 4e,f and Table S4). The GO terms enriched among the 306 genes were related to intermediate filament, intermediate filament cytoskeleton, and polymeric cytoskeletal fibre, which significantly overlapped with the GO terms found in the mutation analysis described in Figure 3. The eQTLs corresponding to downregulated DEGs were enriched in GO terms associated with focal adhesion, establishment of endothelial barrier, angiogenesis, extracellular matrix, macrophage activation and integrin binding functions. We employed a linear regression model to assess the impact of eQTLs on core genes linked with GO outcomes. We examined eQTLs targeting genes related to increased GO functions (Figure 4g,h). Notably, rs2521768 correlates with *KRT* expression variability in AIP patients (*p* value = 1.62 × 10^−6), and rs6952077 affects both *KRTAP2‐1* (*p* value = 1.12 × 10^−6) and *KRTAP24‐1* (*p* value = 3.02 × 10^−6) expression. Regarding genes linked with reduced GO functions, we observed associations of rs3740102 with *FAM107A* (*p* value = 3.20 × 10^−7) and rs2120276 with *AHNAK* (*p* value = 1.12 × 10^−6) and *CLDN5* (*p* value = 3.02 × 10^−6) expression variations in AIP patients.

## 4 Discussion

This study investigates the genomics and transcriptomics of AIP, a type of interstitial lung disease that currently lacks both effective treatments and comprehensive molecular research. Our research elucidates the molecular mechanisms of AIP through detailed genomic and transcriptomic profiling, offering valuable insights and establishing a groundwork for future therapeutic strategies.

In this study, we identified upregulated genes in AIP were enriched for functions related to type I interferon receptor binding. Recent research has reported the significant role of Type I Interferons (IFN‐Is) in the progression of inflammatory diseases by modulating related signalling pathways. Interferons (IFN‐Is) play a regulatory role in inflammation, influencing it through several downstream signalling pathways [18]. To treat acute/subacute interstitial pneumonia, corticosteroids have traditionally been the primary treatment option [19]. Recent research indicates that corticosteroids downregulate Type I interferon (IFN)‐related genes [20]. However, there is a need for more detailed studies to understand the specific molecular targets and mechanisms by which corticosteroids influence type I IFN‐related immunosuppression. Another set of genes, exhibiting upregulated expression differences between AIP patients and controls, was found to be enriched in pathways associated with keratin and intermediate filament functions. Keratin proteins are the primary type of intermediate filaments (IFs) in epithelial tissues and make up about three‐quarters of the IF superfamily. In cases of human diseases, there are dynamic changes in the expression levels and posttranslational modifications of keratins. For instance, diseases such as inflammatory disorders often feature elevated levels of keratin expression. This overexpression can disrupt the balance of keratin ratios, potentially leading to the formation of aggregates [21]. Significantly upregulated genes in AIP, associated with Type I interferon and keratin, may indicate potential for effective targeted blockers that bypass corticosteroid effects used in treatment, while also highlighting the need for further investigation into keratin overexpression.

We found that genes related to focal adhesion and endothelial barrier integrity in AIP, such as FAM107A, TNS1, TNS2, PPP1R16B, PECAM1 and CLDN5. Focal adhesions play a crucial role in lung health by controlling endothelial barrier permeability, essential for gas exchange [22, 23]. The observed downregulation suggests disruptions in lung structural integrity and cellular functions like adhesion, migration, and mechanical sensing [24]. Specifically, TNS1 and TNS2 are linked to fibronectin and collagen matrix assembly and Rho activity regulation, essential for tissue remodelling [25, 26]. Similarly, PPP1R16B, PECAM1 and CLDN5 are vital for maintaining endothelial barrier function, with their downregulation indicating a compromised barrier, potentially impacting AIP pathophysiology [27, 28, 29, 30]. This underscores the importance of the endothelial barrier in AIP and highlights potential therapeutic targets for addressing the disease's underlying mechanisms.

Idiopathic interstitial pneumonias (IIPs) include a variety of lung disorders characterised by differing inflammation and fibrosis levels [31]. Acute Interstitial Pneumonia (AIP) stands out for its rapid onset and progression to respiratory failure, similar to ARDS, but occurring spontaneously. Unlike other IIPs, AIP has been less explored on a molecular level, underscoring the importance of our study which aims to fill this gap by identifying AIP‐specific differentially expressed genes (DEGs) using the GSE213001 dataset. Our findings reveal 2790 upregulated and 3187 downregulated genes specific to AIP, with significant roles in type I interferon receptor binding, keratin filament pathways, and the maintenance of the endothelial barrier, highlighting the molecular uniqueness of AIP within the IIP spectrum.

Our data indicate that coding variants observed in AIPs exhibit some overlap with functions that are enriched in transcriptomic profiling. The genes we focused on as coding variants of AIP are *MUC17*, *UMODL1*, and FN1, which are enriched in the apical part of cell and extracellular matrix. Compared to Korean gnomAD data, the minor allele frequencies are less than 0.05, which can be considered meaningful variants with the potential to cause disease. *MUC17* is one of the membrane mucin groups, the membrane mucins are located in the ductal surfaces of organs epithelial cells served as a physical barrier [32]. Mutations in *MUC17* are known to be associated with poor prognosis in glioma [33]. The *UMODL1* proteins, which are likely to be secreted, are associated with extracellular matrix proteins involved in cell‐to‐cell and cell‐to‐extracellular matrix adhesion. Additionally, *UMODL1* has been reported to have a presence in lung adenocarcinoma and associated with metastasis [34, 35, 36]. Fibronectin 1 (*FN1*) is a critical glycoprotein in the extracellular matrix (ECM), known for its diverse roles in cellular processes like development, adhesion, and wound healing, primarily via integrin signalling [37]. *FN1* mutations can affect different domains of the protein, impacting its interaction with cells and fibrillogenesis [38]. In addition, we observed variants occurring in keratin genes. Several studies shows that mutations in the most conserved regions of keratins have more significant impacts than the complete loss of keratin, disrupting the heteropolymeric network [39]. However, the link between keratin proteins and lung diseases, including AIP, remains unexplored [40].

We identified several cis and trans eQTLs operating in AIP patients, particularly a regulatory network affecting genes related to intermediate filament, focal adhesion and endothelial barrier, and ECM. These eQTL loci affect the expression of keratin‐related genes *KRT37*, *KRTAP2‐1*, *KRTAP24‐1*, focal adhesion‐related genes *FAM107A*, *AHNAK*, and endothelial barrier‐related *CLDN5*. The current shortfall in omics studies on AIP precludes the confirmation of relevant eQTL loci and impacted genes, necessitating further exploration through expansive future analyses.

Our study on AIP acknowledges certain limitations, including the small sample size of the control and AIP groups, as well as the fact that the DEG analyses were conducted without multiple testing correction (Table S5). Furthermore, while the comparison with IPF provides investigative context, these two studies cannot be directly compared due to differences in their control groups, indicating the need for further investigations to validate the identified targets. Despite this, to the best of our knowledge, our research is among the first comprehensive studies using genomic and transcriptomic analysis in AIP. This research provides insights for understanding AIP and identifies potential therapeutic targets, supported by expression profiling, variant profiling and eQTL analysis. Our comparative study involving IPF and non‐IPF ILDs revealed distinct overexpression of type I interferon and keratin function genes, alongside reduced expression of genes related to focal adhesion and endothelial barrier formation in AIP patients. We hope our pioneering research will reveal novel insights and direct future treatment strategies for AIP.

## Author Contributions

**Yun Hak Kim:** conceptualization (lead), investigation (lead), methodology (lead), writing – review and editing (lead). **Donghee Kwak:** conceptualization (lead), formal analysis (lead), investigation (lead), visualization (lead), writing – original draft (lead). **Junho Kang:** conceptualization (lead), formal analysis (equal), investigation (lead), writing – review and editing (equal). **Yeuni Yu:** formal analysis (equal), investigation (equal). **Hansong Lee:** formal analysis (equal), investigation (equal). **Yeongjoo Kim:** formal analysis (equal), investigation (equal). **Eun Jung Kwon:** formal analysis (equal), investigation (equal). **Dong Min Lim:** formal analysis (equal), investigation (equal). **Seongik Mun:** formal analysis (equal), investigation (equal). **Hyo Min Kim:** formal analysis (equal), investigation (equal). **Hae Seul Lee:** formal analysis (equal), investigation (equal). **Hye Ju Yeo:** conceptualization (lead), investigation (lead), writing – review and editing (lead). **Woo Hyun Cho:** conceptualization (lead), investigation (lead), writing – review and editing (lead).

## Ethics Statement

The institutional review board of Pusan National University Yangsan Hospital approved this study (IRB no 05‐2021‐137). All tissue from the biobank were anonymized, and the need for informed consent was waived. All methods were performed in accordance with the relevant guidelines and regulations.

## Conflicts of Interest

The authors declare no conflicts of interest.

## Supporting information


Figure S1.
**Figure S2**.
**Table S1**.
**Table S2**.

Table S3.

Table S4.

Table S5.

## Data Availability

The bulk RNA‐seq data generated in this study have been deposited in the Gene Expression Omnibus (GEO) under accession number GSE283072. WGS data are available in the Sequence Read Archive (SRA) under BioProject accession number PRJNA1192739.
