# Supplementary material for: Integrative Genomic and Transcriptomic Analysis in Acute Interstitial Pneumonia
Source: J Cell Mol Med. 2024 Dec 5;28(23):e70252. doi: 10.1111/jcmm.70252 (PMC11619155; doi:10.1111/jcmm.70252)
Supplement: Supplementary file 1 — Figure S1. Figure S2. Table S1. Table S2. [file JCMM-28-e70252-s004.docx]

Table S1. Baseline characteristics of patients

| Variables | AIP (N=15) |
| --- | --- |
| Age | 55 [49-59] |
| Male, N (%) | 7 (46.7) |
| BMI | 22 [20-27] |
| Never-smoker | 12 (80) |
| Former smoker | 3 (20) |
| Pack-year | 0 [0-0] |
| Pathology |  |
| Diffuse alveolar damage | 15 (100) |

Data presented as median [IQR] or N(%).

Table S2. Baseline characteristics of healthy controls

|  | Control 1 | Control 2 | Control 3 | Control 4 | Control 5 |
| --- | --- | --- | --- | --- | --- |
| Age | 48 | 34 | 18 | 38 | 48 |
| Sex | Male | Female | Male | Male | Male |
| Height, cm | 172 | 167 | 177 | 165 | 178 |
| Weight, kg | 69 | 44 | 80 | 56 | 85 |
| Smoking history | No smoker | No smoker | No smoker | Current smoker | Current smoker |
| Pack* year |  |  |  | 0.5*10 | 0.5*15 |
| Hypertension | - | - | - | - | + |
| Diabetes | - | - | - | - | - |

Fig S1. Group Wilcoxon test comparison of core genes for type I interferon receptor binding and keratin filament (p_value < 0.01 : **, p_value < 0.05 : *, non-significant : ns)

Fig S2. Group Wilcoxon test comparison of core genes for focal adhesion and establishment of endothelial barrier (p_value < 0.001 : ***, p_value < 0.01 : **, p_value < 0.05 : *, non-significant : ns).
